# Effectiveness of mindfulness-based interventions on mental health in natural menopause: a systematic review and meta-analysis

**DOI:** 10.3389/fgwh.2026.1830813

**Published:** 2026-07-10

**Authors:** Flavia Bonfadini, Laura Paplauskaite, Michelle Hughes, Ruimin Ma, Mark Kennedy

**Affiliations:** 1 Institute of Psychiatry, Psychology and Neuroscience (IoPPN), King’s College London, London, United Kingdom; 2 Department of Psychological Medicine, Institute of Psychiatry, Psychology and Neuroscience, King’s College London, London, United Kingdom; 3 Department of Child and Adolescent Psychiatry, Institute of Psychiatry, Psychology and Neuroscience, King’s College London, London, United Kingdom

**Keywords:** anxiety, cognitive health, depression, menopause, mental health, mindfulness

## Abstract

**Background:**

There are currently more than 750 million women worldwide aged 40–60 years, the age during which the majority undergo menopausal transition. This period is often marked by challenging symptoms that affect physical and mental health, productivity, and quality of life, sometimes becoming severe enough to compromise personal and professional relationships. A substantial proportion of women experience mental health symptoms such as anxiety, depression and cognitive complaints. Mindfulness-based interventions (MBIs) have shown promising results in alleviating menopausal symptoms and represent a safe, self-managing alternative or complement to pharmacological treatments.

**Objectives:**

To consolidate and review the current state of the literature on the effectiveness of MBIs for mental health symptoms, including anxiety, depression, and cognitive function in women undergoing natural menopause.

**Methods:**

This systematic review was conducted in accordance with the Preferred Reporting Items for Systematic Reviews and Meta-Analyses (PRISMA) guidelines. It included randomized and non-randomized controlled trials of MBIs in women undergoing natural menopause. The outcomes of interest were mental health, including anxiety, depression, and cognitive function. Databases searched included PubMed, Embase, PsycInfo, CINAHL, and Web of Science. Searches were performed up to October 2025. Reference lists and specialist journals were also hand searched. The methodological quality and risk of bias of the included studies were assessed using the Cochrane Risk of Bias 2.0 (RoB 2) and the Newcastle-Ottawa Scale (NOS). An exploratory random-effects meta-analysis was conducted where measurement consistency permitted.

**Results:**

Ten studies (eight randomized controlled trials and two quasi-experimental designs), including a total of 1,270 participants, met the inclusion criteria. Six studies assessed psychosocial quality of life using the MENQOL Psychosocial Domain, enabling meta-analysis; a large pooled effect favoured MBIs (Hedges' *g* = −1.06, *p* < 0.001). Secondary outcomes of anxiety and depression, measured independently, showed smaller effects. No study assessed cognitive function using validated cognitive instruments.

**Conclusions:**

Preliminary evidence suggests mindfulness-based interventions may improve mental health symptoms during natural menopause. The discrepancy between composite psychosocial effects and standalone mood effects may reflect measurement differences between menopause-specific and generic instruments, or suggest that cognitive complaints contribute to the observed benefits. However, substantial methodological limitations temper confidence in these findings. Future research should prioritise validated cognitive assessment as an independent outcome, investigation of practice dose required to maintain benefits, and comparative effectiveness trials against established treatments.

## 1 Introduction

Globally, more than 750 million women are aged between 40 and 60 years (1), the age group most commonly experiencing the menopausal transition. This period is characterized by endocrine changes that lead to vasomotor, somatic, and psychosocial symptoms, many of which can be severe enough to impair daily life (2). Approximately 22% of women experience clinically significant anxiety (3) and 35% experience depression (4) during this period. Subjective cognitive difficulties are also highly common, with 44%–62% of women reporting cognitive decline during the menopausal transition in population-based studies (5, 6), though these symptoms remain inadequately studied in intervention research. Together, these symptoms substantially reduce quality of life (7).

The burden of menopause is not only personal but also societal. Reduced work performance, absenteeism, and greater demand on healthcare services represent growing economic and public health challenges (8, 9). As life expectancy rises and the proportion of older women increases globally, the challenges associated with menopause are expected to intensify (1).

Standard treatments for menopausal symptoms include pharmacological approaches such as hormone replacement therapy (HRT) and antidepressants, alongside psychotherapeutic approaches such as cognitive behavioral therapy (CBT) (10). While these interventions can be effective, they are often limited by side effects, contraindications, long-term safety concerns, accessibility issues, or adherence problems (11). These limitations have prompted increasing interest in safe, self-directed, non-pharmacological strategies that enable women to manage their symptoms more independently.

Among these, Mindfulness-Based Stress Reduction (MBSR) and Mindfulness-Based Cognitive Therapy (MBCT) have emerged as promising approaches. Unlike broader mindfulness-related approaches, MBSR and MBCT are the most extensively validated MBI protocols, with established curricula, standardised delivery formats, and a substantial evidence base demonstrating improvements in mental health across clinical populations (12). Evidence from mindfulness studies of different populations indicates that MBIs can improve anxiety, depression and cognitive functioning, while enhancing overall mental health and quality of life (13). Mindfulness is low-cost, adaptable across settings, and carries minimal risk, offering a promising non-pharmacological approach for menopausal women.

Despite the growing body of research on mindfulness in mental health, evidence on MBIs in menopause is scarce, with only two reviews focusing on outcomes that are similar to our study (14, 15). However, these reviews included broader mindfulness-related interventions rather than exclusively examining established protocols such as MBSR and MBCT, making direct comparison of findings difficult. Furthermore, while one of those reviews by Wang and colleagues (15), separately calculated MENQOL Psychosocial Domain as one of its numerous outcomes measures, they did not evaluate it in depth as a distinct outcome domain. No review to date has examined this domain, which incorporates cognitive complaints alongside mood-related symptoms, in relation to separately measured anxiety and depression outcomes, considered the implications of this composite structure, or explored the durability of these effects across follow-up periods.

The present systematic review addresses these gaps by examining the effectiveness of MBSR & MBCT on mental health in women undergoing natural menopause, with the MENQOL Psychosocial Domain as the primary outcome and anxiety and depression as secondary outcomes. This dual approach enables comparison between composite psychosocial effects and independently measured mood outcomes, raising important questions about what drives the observed psychosocial benefits and underscoring the need for future studies employing validated cognitive assessment in menopausal populations.

## 2 Methods

This systematic review was conducted in accordance with the Preferred Reporting Items for Systematic Reviews and Meta-Analyses (PRISMA) 2020 guidelines (16).

### 2.1 Search strategy

**Figure 1:**
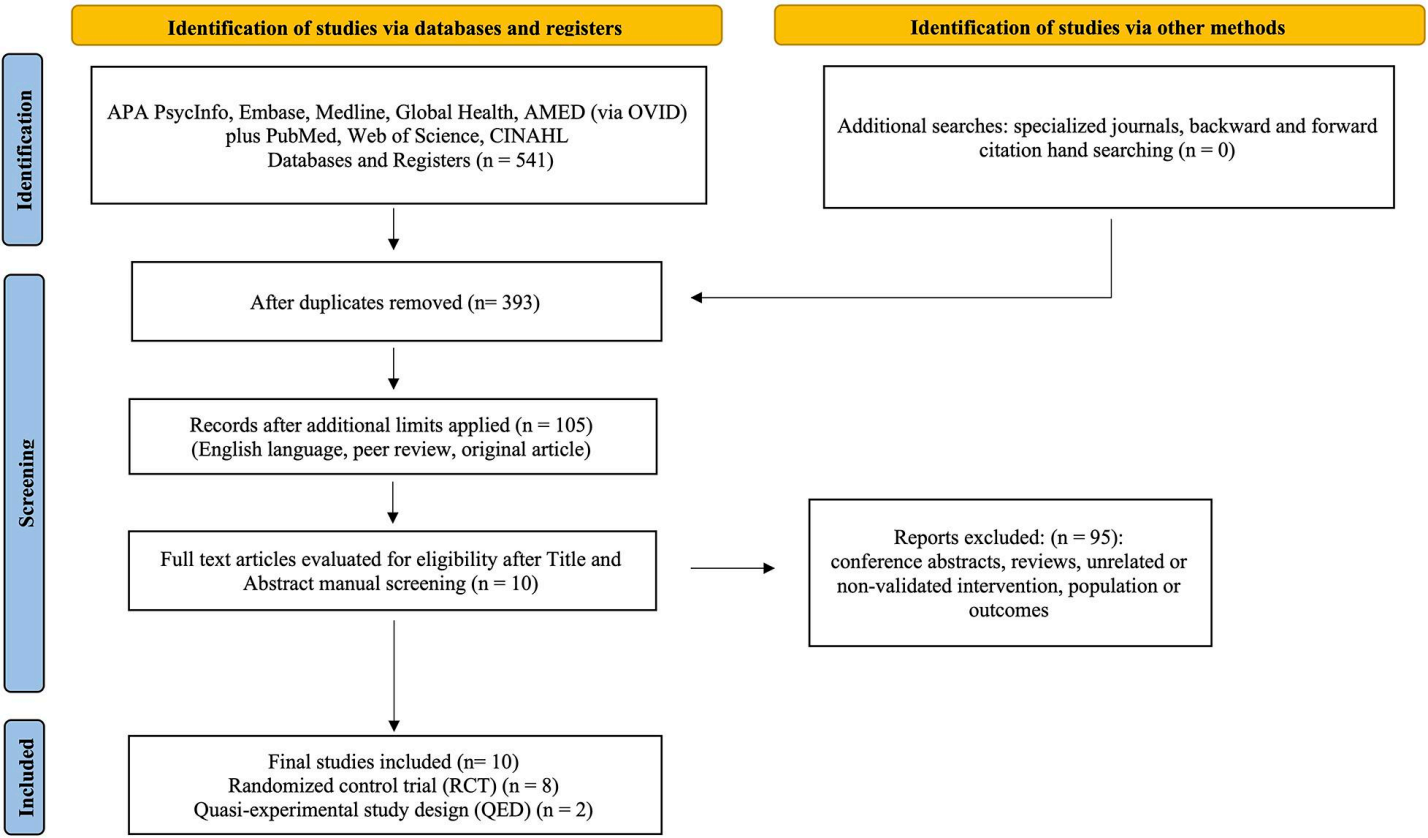
Source: Page MJ, et al. BMJ 2021;372:n71. doi: 10.1136/bmj.n71. This work is licensed under CC BY 4.0. To view a copy of this license, visit https://creativecommons.org/license/by/4.0.

A literature search was performed by 3 independent reviewers across multiple electronic databases. The initial search used a string consisting of the keywords “*mindfulness*” and “*menopause*” entered into APA PsycInfo, Embase, Medline, Global Health, AMED, PubMed, Web of Science, and CINAHL. The search strategy used a PICOS structure without specifying comparators, outcomes, or date limits to maximize inclusion and was last updated in October 2025. Subsequent searches expanded the criteria incorporating MeSH terms and outcome-related keywords (mindful* OR mindfulness?based* OR MBI* OR MBCT OR MBSR) AND (menopaus* OR *menopaus* OR premenopaus* OR perimenopaus* OR postmenopaus* OR climacteric). Specialized journals (Mindfulness, Mindfulness & Compassion, Climacteric, Journal of Menopausal Medicine, Journal of Midlife Health, Maturitas, Menopause, and Post Reproductive Health Journal) were also searched. Hand-searching was performed through reference lists and backward and forward citations. Figure 1 illustrates the process, resulting in 10 publications. The study protocol was not prospectively registered in PROSPERO or another systematic review registration platform.

### 2.2 Literature inclusion and exclusion criteria

The eligibility criteria were structured using the PICOS framework. Population: Women experiencing natural menopause, including peri and postmenopausal stages; Intervention: Validated mindfulness-based interventions (MBIs): MBSR and MBCT; Comparison: Any comparator was eligible, including active treatments, waitlist controls, treatment-as-usual, or no-treatment/placebo groups; Outcomes: Mental health - anxiety, depression, and cognitive function - assessed using validated measurement instruments. Study Design: Intervention trials (randomized or non-randomized) reporting quantitative outcome data. Peer-reviewed papers published in English were eligible for inclusion. Conference abstracts, cross-sectional studies, reviews, grey literature, and studies including surgically induced menopause participants and reporting symptoms unrelated to specified outcomes were excluded.

### 2.3 Screening

After the literature search, deduplication was conducted, followed by title and abstract screening, and full-text review. Screening was conducted by 3 independent reviewers, with any disagreements resolved through discussion.

### 2.4 Risk of bias and quality assessment of studies

Risk of bias was assessed independently by 3 reviewers (FB, LP, and MH) using the Cochrane Risk of Bias 2.0 (RoB 2) tool for randomized controlled trials (RCTs) (17) and the Newcastle-Ottawa Scale (NOS) for quasi-experimental studies (QEDs) (18). Detailed RoB 2 and NOS assessments are presented in Tables 1 and 2, respectively. Disagreements were resolved through discussion between the reviewers until a consensus was reached.

**Table 1:** Risk of bias assessment (RoB 2 - RCTs).

Author, Year	Randomization process	Deviations from intended interventions	Missing outcome data	Measurement of the outcome	Selection of the reported result	Overall bias
Carmody et al. (25) (2011)	Low	Low	Some concerns	High	Some concerns	High
Enjezab et al. (21) (2019)	Some concerns	Low	Some concerns	High	Some concerns	High
Gordon et al. (24) (2021)	Low	Low	Low	High	Some concerns	High
Huang et al. (31) (2023)	Some concerns	Low	Low	Low	Some concerns	Some concerns
John et al. (20) (2022)	Low	Low	Low	High	Some concerns	High
Koca et al. (32) (2024)	Some concerns	Low	Low	High	Some concerns	High
Wong et al. (28) (2018)	Low	Low	Low	Low	High	High
Yazdani Aliabadi et al. (30) (2021)	Low	Low	Some concerns	High	Some concerns	High

**Table 2:** Quality assessment (NOS - Non-RCTs).

Author, Year	Representativeness of the exposed cohort	Selection of the non-exposed cohort	Ascertainment of exposure	Outcome of interest was not present at start of study	Comparability of cohorts on the basis of the design or analysis	Assessment of outcome	Was follow-up long enough for outcomes to occur	Adequacy of follow up of cohorts	Total (9/9)
Şener & Timur Taşhan. (29) (2021)	*	0	*	*	*	0	*	0	5/9
Şener Çetin et al. (23) (2024)	*	0	*	*	*	0	*	0	5/9

* indicates criterion met (1 point awarded); 0 indicates criterion not met.

### 2.5 Statistical analysis

Statistical analyses were conducted using JASP (19). Meta-analysis was performed for psychosocial outcomes measured using the MENQOL Psychosocial Domain, as six studies reported sufficiently comparable outcome data. Given apparent differences in MENQOL scoring approaches across studies, effect sizes were calculated as standardized mean differences using Hedges' g. Change-score effects were calculated from baseline to post-intervention for intervention and control groups. Where standard deviations of change were not reported, they were estimated from baseline and post-intervention standard deviations using an assumed pre-post correlation of *r* = 0.50. Negative effect sizes indicate greater reductions in psychosocial symptoms in the intervention group compared with control.

A random-effects meta-analysis using restricted maximum likelihood (REML) estimation was conducted to account for expected between-study heterogeneity in intervention type, study design, population characteristics, and scoring approaches. Effects were pooled using the inverse-variance method and reported with 95% confidence intervals and prediction intervals. Heterogeneity was assessed using Cochran's Q and *τ*^2^ statistics.

Sensitivity analyses were conducted to assess the robustness of the pooled effect. These included varying the assumed pre-post correlation used to estimate change-score standard deviations (r = 0.30 and r = 0.70), excluding studies using alternative MENQOL scoring approaches [John et al. (20) and Enjezab et al. (21)], and conducting design-based sensitivity analyses comparing pooled effects across all included studies, randomized controlled trials only, and quasi-experimental studies only. Leave-one-out analyses were additionally conducted to assess the influence of individual studies on the pooled effect estimate. Follow-up assessments reported by five studies were additionally examined narratively to assess the durability of psychosocial symptom changes over time. Statistical significance was determined using two-sided *p* < .05.

Publication bias was not formally assessed due to the limited number of included studies (<10), as funnel plot asymmetry and Egger's regression are considered unreliable with small study numbers (22).

## 3 Results

### 3.1 Study selection

Initial search returned 541 results that were reduced to 393 after deduplication. Subsequent searches adding key terms or limiting date ranges returned too few results; therefore, the original search string was maintained. Further applied limits narrowed it down to 105 results. Title, abstract, and full-text screenings excluded 95 papers, resulting in 10 articles reviewed as shown in the PRISMA flow diagram (Figure 1).

### 3.2 Characteristics of participants and study settings

Eight RCTs and two QEDs were included. Participant numbers ranged from 50 in John et al. (20) to 200 in Şener Çetin et al. (23), with a combined total of 1,270 participants recruited and 1,102 analyzed (522 control, 508 mindfulness intervention, and 72 allocated to other intervention arms). The studies were conducted across six countries: two in Iran, three in Turkey, two in China, and one each in the USA, Canada, and India. Detailed study characteristics, recruitment methods, intervention protocols, and study limitations are summarized in Tables 3 and 4.

**Table 3:** Summarized characteristics of studies.

Reference	MBI	Recruited population	Control	Outcomes	Design	Measures
Carmody et al. (25) USA	MBSR	Menopausal women, mixed sample (peri- and post) *n* = 110 (age = 47–69) *n* = 57 (MBSR) *n* = 53 (control)	Waitlist	Primary: hot flush bother; secondary: hot flush intensity, quality of life (outcomes for subdomains not provided), insomnia, anxiety, perceived stress, treatment adherence	RCT	MENQOL, PSS, WHIIRS, HADS-A
Enjezab et al. (21) Iran	MBCT	Perimenopausal women *n* = 83 (age = 45–55) *n* = 42 (MBCT) *n* = 41 (control)	No intervention	Quality of life, including psychosocial symptoms	RCT	MENQOL, KMI
Gordon et al. (24) Canada	MBSR	Perimenopausal women *n* = 104 (age = 45–55) *n* = 52 (MBSR) *n* = 52 (control)	Waitlist	Primary: depressive symptoms; secondary: perceived stress, anxiety, resilience, sleep	RCT	CES-D, DRSP, PSS, STAI, CD-RISC, FFMQ, PSQI, HFRDIS, THQ, LES, DRSP, E1G assays
Huang et al. (31) China	MBSR	Menopausal women *n* = 120 (average age = 59) *n* = 62 (MBSR) *n* = 58 (control)	Active (8-week menopausal education)	Anxiety disorder	RCT	GAD-7, FFMQ, hormonal assays (FSH, E2, 5-HT)
John et al. (20) India	MBCT	Menopausal women *n* = 50 (age = 45–60) *n* = 25 (MBCT) *n* = 25 (control)	No intervention	Quality of life, including psychosocial symptoms	RCT	KMI, MENQOL
Koca et al. (32) Turkey	MBSR	Postmenopausal women *n* = 180 (average age = 55) *n* = 45 (MBSR) *n* = 45 (laughter yoga) *n* = 45 (acupressure) *n* = 45 (control)	No intervention	Menopausal symptoms, quality of life, including psychosocial symptoms	RCT	MRS, MENQOL
Şener Çetin et al. (23) Turkey	MBSR	Postmenopausal women *n* = 200 (age = 45–72) *n* = 100 (MBSR) *n* = 100 (control)	No intervention	Insomnia (sleep), quality of life, including psychosocial symptoms	QED	WHIIRS, MENQOL
Şener & Timur Taşhan (29) Turkey	MBSR	Postmenopausal women *n* = 160 (age = 45–70) *n* = 80 (MBSR) *n* = 80 (control)	No intervention	Quality of life, including psychosocial symptoms	QED	MAAS, MRS, MENQOL
Wong et al. (28) China	MBSR	Menopausal women, mixed sample *n* = 197 (age = 40–60) *n* = 98 (MBSR) *n* = 99 (control)	Active (8-week Menopausal education)	Primary: anxiety, depression, somatic symptoms (urogenital, sexual, VMS); secondary: Perceived stress, mindfulness, quality of life	RCT	GCS, PSS, FFMQ, SF-12
Yazdani Aliabadi et al. (30) Iran	MBSR	Postmenopausal women *n* = 66 (age = 47–62) *n* = 33 (MBSR) *n* = 33 (control)	No intervention	Quality of life, including psychosocial symptoms	RCT	MENQOL

5-HT, 5-hydroxytryptamine; CES-D, center for epidemiologic studies depression scale; CD-RISC, Connor-Davidson resilience scale; DRSP, daily record of severity of problems; E2, estradiol; E1G, estrogen marker; FFMQ, five facet mindfulness questionnaire; FSH, follicle stimulating hormone; GAD-7, generalized anxiety disorder; GCS, modified Greene climacteric scale; HADS-A, hospital anxiety and depression scale-anxiety; HFRDIS, Hot flash related daily interference scale; KMI, Kupperman menopausal index; LES, life events survey; MAAS, mindfulness awareness attention scale; MENQOL, menopause-related quality of life; MRS, menopausal symptoms rating scale; PSQI, Pittsburgh sleep quality index; PSS, perceived stress scale; SF-12, medical outcomes study short-form health survey; STAI, state-trait anxiety inventory; THQ, trauma history questionnaire; VMS, vasomotor symptoms; WHIIRS, women's health initiative insomnia rating scale.

**Table 4:** Additional characteristics of studies.

Reference	Recruitment	Timepoints	Results	Strengths & Limitations
Carmody et al. (25) USA	Direct mail, newspaper, radio, cable; personal and provider referral; medical school intranet and posters; workplace, health fairs, community and menopause events	8 weekly 2 ½-h class Daylong retreat Homework (45 min daily) 3-month follow-up (week 20 from enrollment)	MBSR significantly improved anxiety measures. MBSR may be effective in improving quality of life, sleep, and VSM symptoms' distress and bother, but not intensity.	S: RCT; sample size; homework; retreat; dropout criteria; follow-up L: no active control; non-diverse sample (mostly high socioeconomic, educated White women); self-report
Enjezab et al. (21) Iran	Four local health centers	8 weekly 2-h class No retreat Homework (1-h daily) 1-month follow-up (week 12 from enrollment)	MBCT significantly improved physical and psychosocial symptoms of quality of life.	S: RCT; original study in Iran; homework; follow-up L: no active control; short follow-up; no home practice materials; self-report
Gordon et al. (24) Canada	Social media paid advertising	8 weekly 2 ½-h class Daylong retreat Homework (45 min daily) 6-month follow-up reported only post-intervention mean up to 6-month follow-up (weeks 8–32 from enrollment)	MBSR was effective in improving sleep, resilience, anxiety, and depressive symptoms, with significant results on reducing perceived stress.	S: RCT; retreat; homework; repeated measures; dropout criteria; follow-up; fidelity; examined potential moderators L: no active control, non-diverse (mostly high socio-economic, educated White women); self-selection; online follow-up; did not report post-intervention or 6 -month follow- up results - just the mean.
Huang et al. (31) China	Single-center outpatients	8 weekly 1-h class No retreat No homework No follow-up	MBSR was effective in significantly improving anxiety and hormone levels.	S: RCT; active control; random allocation L: small sample; no follow-up; convenience sampling; no homework
John et al. (20) India	Local hospital outpatients	8 weekly 1-h class No retreat Homework (unspecified) 2-week follow-up (week 10 from enrollment)	MBCT significantly improved quality of life through VMS and psychosocial symptom reduction.	S: RCT; homework; follow-up; dropout criteria L: no active control, short follow-up; convenience sampling; self-report
Koca et al. (32) Turkey	Single family health center	8 weekly 2 ½-h class Daylong retreat (6-h silence day) Homework (repeat class exercises) No follow-up	When measured using MRS, MBSR significantly reduced overall menopause symptoms across all subscales. For quality of life (MENQOL), MBSR only improved psychosocial domain.	S: RCT; one day retreat; homework; L: single center sample; no follow-up; self-report; no active control group
Şener Çetin et al. (23) Turkey	Two separate family health centers	8 weekly 2–2 ½-h sessions 6-h silent retreat Homework 2-month follow-up (week 16 from enrollment)	MBSR increased quality of life, including improving psychosocial symptoms, and decreased insomnia during intervention, but no significant effects on sleep at follow-up.	S: pre-post and mid-tests; motivational messages and reminders for continued practice after intervention; large sample; control group; follow-up L: no active control, QED with samples from only two health centers limits generalization to other populations; no hormonal assessment; no homework during intervention; self-report
Şener & Timur Taşhan (29) Turkey	Two local health centers	8 weekly 2–2½-h class Daylong retreat No homework 2-month follow-up (week 16 from enrollment)	MBSR was significantly effective in improving quality of life, including psychosocial symptoms.	S: pre-post and mid-tests; continued practice after intervention; follow-up L: no active control, QED limits generalization to other populations; no homework; self-report
Wong et al. (28) China	Community and primary care settings: local newspaper advertisements; internal emails to university staff, students, and alumni; women centers’ newsletters; posters and leaflets in clinics	8 weekly 2 ½-h class No retreat Homework (40 min daily) 6-month follow-up (week 32 from enrollment)	MBSR and MEC reduced menopausal symptoms. MBSR was more effective than control and significantly reduced anxiety and depression symptoms; no improvement in somatic symptoms (urogenital and VSM).	S: RCT; largest sample; matched active control; dropout criteria; follow-up; fidelity checks; homework L: non-diverse sample (mostly married, young Chinese women); self-selection; self-report
Yazdani Aliabadi et al. (30) Iran	Mostly local gathering centers	8 weekly 2-h class No retreat Homework (unspecified) 3-month follow-up (week 20 from enrollment)	MBSR was effective and safe in significantly improving quality of life across physical, psychosocial, and sexual domains.	S: RCT; cost-effective; learning materials; dropout criteria; follow-up L: no active control, non-diverse sample (mostly non-working Persian women); self-report

Age ranged from 40 to 72 years, with mean ages spanning from 48.7 years in Gordon et al. (24) to 56.6 years in Şener Çetin et al. (23) Menopausal stages included perimenopausal (*n* = 2), postmenopausal (*n* = 4), menopause (*n* = 2), and mixed stages (*n* = 2). Definitions of menopausal status varied considerably across studies. Only Gordon et al. (24) and Carmody et al. (25) explicitly applied staging frameworks - the STRAW+10 (26) and original STRAW criteria (27), respectively. Wong et al. (28) used operationally consistent definitions based on menstrual pattern changes without naming a formal framework. Three postmenopausal studies (Şener Çetin et al. (23); Şener & Timur Taşhan (29); Yazdani Aliabadi et al. (30) defined postmenopause as absence of menstruation for 12 months or more. The remaining studies used varying approaches: Huang et al. (31) applied Chinese national clinical diagnostic criteria with hormonal confirmation, while John et al. (20) and Enjezab et al. (21) used a Kupperman's index threshold (≥15) confirming symptom severity but not menopausal staging. Koca et al. (32) did not specify diagnostic criteria.

### 3.3 Characteristics of interventions

Among the included MBIs, weekly sessions ranged from 1 to 2.5 h over eight weeks, with some studies incorporating a full-day retreat. Eight studies examined MBSR, generally following Kabat-Zinn's (33) traditional curriculum, including body scan, sitting meditation, gentle mindful movement, breathwork, and group discussion. Two studies examined MBCT, which additionally incorporated mindful walking and mindful eating practices. Groups met at community centers and healthcare facilities.

### 3.4 Characteristics of outcome measurement scales

The included studies employed a range of validated instruments to assess mental health outcomes. Six studies investigated psychosocial symptoms, four examined anxiety and two assessed depression (Tables 5–7).

**Table 5:** Study outcomes - psychosocial symptoms.

Author, Year	Analyzed sample (intervention v control)	Outcome measure	Baseline measure	Post-intervention measure	Follow-up measure	Effect sizes
Enjezab et al. (21) (2019)	MBCT (36) v no intervention control (37)	MENQOL Psychosocial Domain	Intervention: 2.55 (SD = 1.45) Control: 2.02 (SD = 1.15)	Intervention: 1.06 (SD = 0.78) Control: 2.26 (SD = 0.96) *p* < .001	Intervention: 1.28 (SD = 0.84) Control: 2.32 (SD = 0.92) *p* < .001 1-month follow-up (week 12 from enrollment)	Not reported
John et al. (20) (2022)	MBCT (25) v no intervention control (25)	MENQOL Psychosocial Domain	Intervention: 2.80 (SD = 0.81) Control: 2.88 (SD = 1.26)	Intervention: 0.70 (SD = 0.32) Control: 2.20 (SD = 0.72) *p* < .001	Intervention: 0.56 (SD = 0.17) Control: 1.82 (SD = 0.68) *p* < .001 2-week follow-up (week 10 from enrollment)	Not reported
Koca et al. (32) (2024)	MBSR (37) v no intervention control (37)	MENQOL Psychosocial Domain	Intervention: 23.67 (SD = 10.54) Control: 26.65 (SD = 9.17)	Intervention: 19.23 (SD = 7.18) Control: 28.08 (SD = 8.78 *p* < .001	Not followed up	Not reported
Şener Çetin et al. (23) (2024)	MBSR (73) v no intervention control (78)	MENQOL Psychosocial Domain	Intervention: 24.61 (SD = 9.25) Control: 23.06 (SD = 9.94)	Intervention: 17.15 (SD = 6.94) Control: 23.51 (SD = 9.74) *p* < .001	Intervention: 18.46 (SD = 7.57) Control: 23.67 (SD = 9.52) *p* < .001 2-month follow-up (week 16 from enrollment)	Not reported
Şener & Timur Taşhan (29) (2021)	MBSR (55) v no intervention control (63)	MENQOL Psychosocial Domain	Intervention: 25.38 (SD = 11.96) Control: 23.08 (SD = 10.13)	Intervention: 16.96 (SD = 7.15) Control: 23.40 (SD = 10.14) *p* < .001	Intervention: 17.98 (SD = 8.21) Control: 23.67 (SD = 9.84) *p* = .003 2-month follow-up (week 16 from enrollment)	Not reported
Yazdani Aliabadi et al. (30) (2021)	MBSR (30) v no intervention control (30)	MENQOL Psychosocial Domain	Intervention: 22.53 (SD = 6.99) Control: 21.66 (SD = 6.86)	Intervention: 16.20 (SD = 4.95) Control: 24.10 (SD = 6.29) *p* < .001	Intervention: 16.76 (SD = 5.26) Control: 23.83 (SD = 6.56) *p* < .001 3-month follow-up (week 20 from enrollment)	Not reported

MENQOL, menopause-specific quality of life scale. Effect sizes were not reported in the original studies. MBCT studies [Enjezab et al. (21); John et al. (20)] reported mean item scores, while MBSR studies reported summed total scores for the MENQOL Psychosocial Domain. Higher scores indicate greater symptom severity and poorer quality of life.

**Table 6:** Study outcomes – anxiety.

Author, Year	Analysed sample (intervention v control)	Outcome measure	Baseline measure	Post-intervention measure	Follow-up measure	Effect sizes
Carmody et al. (25) (2011)	MBSR (48) v waitlist control (44)	HADS-A	Intervention: 9.6 (SD = 4.3) Control: 7.4 (SD = 3.3)	Change from baseline: Intervention: −3.05 (SE = 0.53) Control: −0.80 (SE = 0.54) *p* = .005	Change from post-intervention: Intervention: +0.65 (SE = 0.51) Control: +0.26 (SE = 0.50) *p* = .586 3-month follow-up (week 20 from enrollment)	Not reported
Gordon et al. (24) (2021)	MBSR (44) v waitlist control (51)	STAI	Intervention: 40.0 (SD = 10.1) Control: 36.8 (SD = 9.4)	Not reported	Intervention: 33.9 (SE = 0.6) Control: 39.3 (SE = 0.5) *p* < .001 Post-intervention mean up to 6-month follow-up (weeks 8–32 from enrollment)	Cohen's d = 0.53 (moderate)
Huang et al. (31) (2023)	MBSR (62) v active control (58)	GAD-7	Intervention: 14.39 (SD = 2.01) Control: 14.28 (SD = 2.16)	Intervention: 8.13 (SD = 1.82) Control: 11.26 (SD = 2.56) *p* = <.001	Not followed up	Not reported
Wong et al. (28) (2018)	MBSR (98) v active control (99)	GCS Anxiety Component	Intervention: 8.23 (SD = 2.96) Control: 8.00 (SD = 2.48)	Not reported	Intervention: 5.97 (SD = 2.49) Control: 6.97 (SD = 2.57) 6-month follow-up (week 32 from enrollment)	Cohen's *d* = 0.46 (moderate) Between-group difference: −0.99 (95% CI: −1.71 to −0.28) Complete case: *p* = 0.007 ITT: *p* = .070

GAD-7, generalized anxiety disorder 7-item scale (range 0–21); GCS, Greene climacteric scale (anxiety component range 0–21); HADS-A, hospital anxiety and depression scale-anxiety subscale (range 0–21); STAI, state-trait anxiety inventory (range 20–80). Higher scores indicate greater anxiety symptoms on all measures. Effect sizes were reported by two of four studies.

**Table 7:** Study outcomes – depression.

Author, Year	Analysed sample (intervention v control)	Outcome measure	Baseline measure	Post-intervention measure	Follow-up measure	Effect sizes
Gordon et al. (24) (2021)	MBSR (44) v waitlist control (51)	CES-D	Intervention: 8.9 (SD = 7.0) Control: 10.1 (SD = 7.7)	Not reported	Intervention: 9.1 (SE = 0.4) Control: 12.4 (SE = 0.3) *p* < .001 Post-intervention mean up to 6-month follow-up (weeks 8–32 from enrollment)	Cohen's *d* = 0.34 (small to moderate)
Wong et al. (28) (2018)	MBSR (98) v active control (99)	GCS Depression Component	Intervention: 6.96 (SD = 2.95) Control: 6.59 (SD = 2.45)	Not reported	Intervention: 4.78 (SD = 2.60) Control: 5.39 (SD = 2.41) 6-month follow-up (week 32 from enrollment)	Cohen's *d* = 0.24 (small) Between-group difference: −0.77 (−1.47 to −0.07) *p* = .031

CES-D, center for epidemiologic studies depression scale (range 0–60); GCS, Greene climacteric scale (depression component range 0–21). Higher scores indicate greater depressive symptoms on both measures.

No study measured cognitive complaints as an independent outcome. However, psychosocial symptoms were consistently assessed using the Menopause-Specific Quality of Life Scale (MENQOL) Psychosocial Domain. The MENQOL Psychosocial Domain comprises seven items: feeling dissatisfied with personal life, experiencing anxiety or nervousness, poor memory, accomplishing less than usual, feeling depressed or blue, being impatient with others, and desiring to be alone (34). Of these, five items substantially overlap with anxiety and depression constructs, while two - poor memory and accomplishing less than usual - capture cognitive difficulties. Given the absence of direct cognitive measures and the inclusion of cognitive complaint items within this domain, MENQOL Psychosocial scores provided the only available indicator of cognitive difficulties in the included studies, though cognitive-specific effects cannot be isolated from the composite measure.

Anxiety measures varied across studies and included the Hospital Anxiety and Depression Scale-Anxiety (HADS-A), State-Trait Anxiety Inventory (STAI), Generalized Anxiety Disorder 7-item scale (GAD-7), and the Greene Climacteric Scale (GCS) Anxiety Component. Depression was assessed using the Center for Epidemiologic Studies Depression Scale (CES-D) and the GCS Depression Component.

Anxiety and depression outcomes were synthesized narratively due to substantial variation in measurement instruments and outcome classification approaches across studies. Previous reviews have also already quantitatively synthesized these outcomes across broader mindfulness-related interventions (14, 15). In contrast, a meta-analysis was conducted for psychosocial outcomes measured using the MENQOL Psychosocial Domain, as six studies reported sufficiently comparable data. The present review specifically focused on established mindfulness-based interventions (MBSR and MBCT) and examined the MENQOL Psychosocial Domain as a distinct composite outcome encompassing cognitive complaints alongside mood-related symptoms.

### 3.5 Result outcomes - psychosocial symptoms

**Figure 2:**
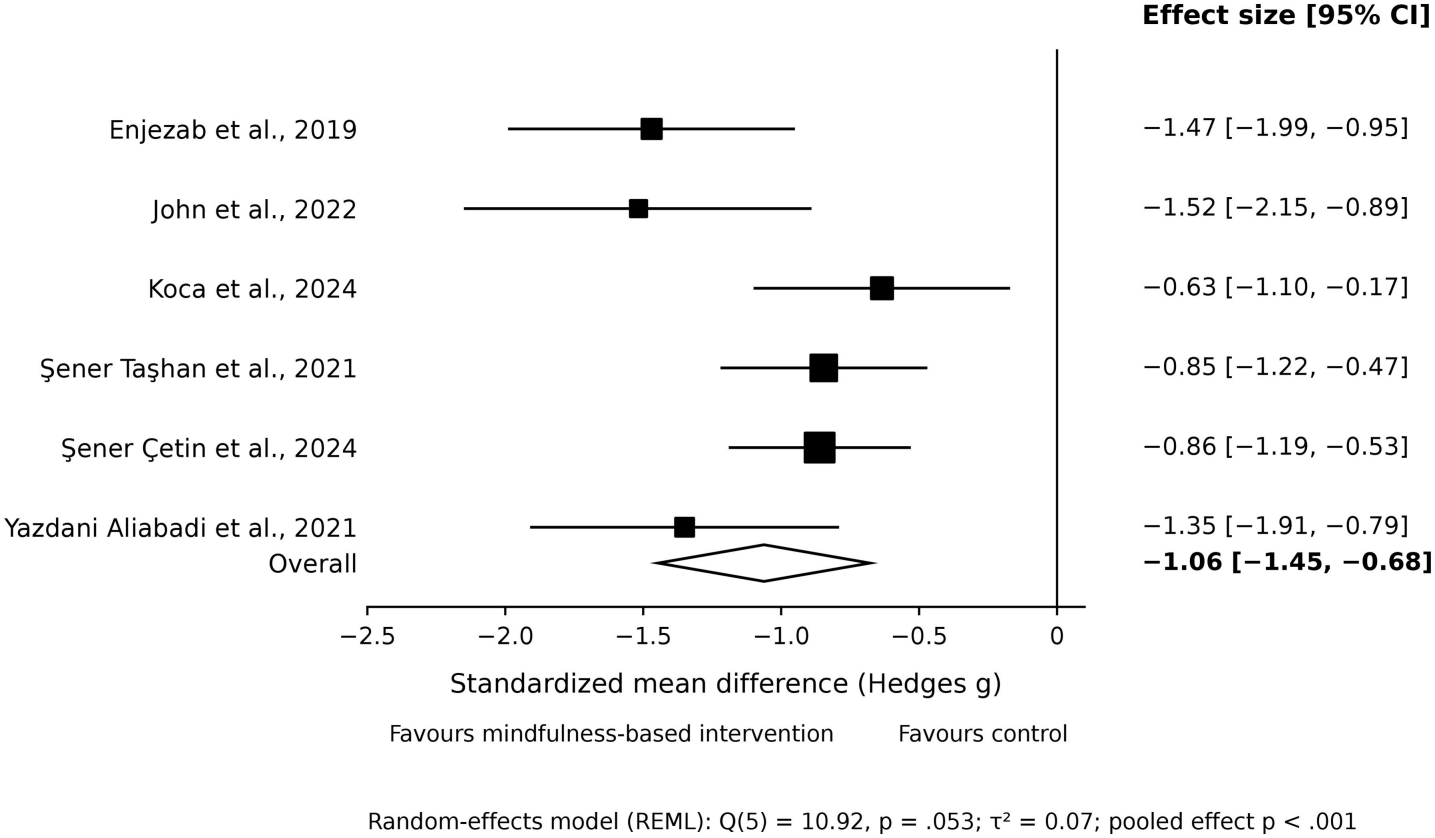
Forest plot: effects of mindfulness-based intervention on psychosocial outcomes measured using the MENQOL pyschosocial domain.

Six studies examined psychosocial symptoms using the MENQOL Psychosocial Domain, which assesses cognitive complaints, anxiety, and depressive symptoms (Table 5). All six studies used control groups with no interventions. Two studies examined MBCT (20, 21), and four examined MBSR interventions (23, 29, 30, 32). The two MBCT studies reported substantially lower numerical values (range 0.56–2.88) compared to the four MBSR studies (range 16.20–28.08), suggesting different scoring approaches (likely mean item scores vs. summed total scores, respectively), though this could not be definitively confirmed from the reported data. Consequently, direct numerical comparisons between MBCT and MBSR study findings should be interpreted with caution. Random-effects meta-analysis demonstrated a significant reduction in psychosocial symptoms following mindfulness-based interventions compared with control conditions [Hedges’ g = −1.06, 95% CI (−1.45, −0.68), *p* < .001; Figure 2]. Heterogeneity was moderate and approached statistical significance [Q(5) = 10.92, *p* = .053; *τ*^2^ = 0.07]. The 95% prediction interval remained below zero (−1.84 to −0.28).

Across all included studies, intervention groups demonstrated reductions in psychosocial symptom scores following MBCT or MBSR interventions, whereas control group scores generally remained stable or increased over time. Five studies included follow-up assessments ranging from two weeks to three months post-intervention, with sustained reductions in psychosocial symptoms observed across all follow-up periods.

Sensitivity analyses demonstrated that the direction and statistical significance of the pooled effect remained consistent when varying the assumed pre-post correlation (*r* = 0.30 and *r* = 0.70), excluding studies using alternative MENQOL scoring approaches, restricting analyses to randomized controlled trials only, and conducting leave-one-out analyses (Supplementary Figure S1). Although effect size magnitude and heterogeneity estimates varied modestly across models, all analyses continued to demonstrate beneficial effects of mindfulness-based interventions on psychosocial symptoms.

A secondary pooled analysis of follow-up data from five studies similarly demonstrated significant reductions in psychosocial symptoms following mindfulness-based interventions compared with control conditions [Hedges' *g* = −0.99, 95% CI (−1.36, −0.63), *p* = .002; Supplementary Figure S2]. Heterogeneity was lower and non-significant in the follow-up analysis [Q(4) = 6.49, *p* = .166; *τ*^2^ = 0.04], and the prediction interval remained below zero (−1.64 to −0.35).

### 3.6 Result outcomes - anxiety

As a secondary outcome, anxiety was examined in four different studies using different measurement instruments (Table 6), which limits direct numerical comparison of results across studies.

Carmody et al. (25) demonstrated a significant reduction in anxiety symptoms post-intervention on the HADS-A scale, with the MBSR group showing a change from baseline of −3.05 compared to −0.80 in the waitlist control group (*p* = .005). At three-month follow-up (week 20 from enrollment), both groups showed small increases from their post-intervention levels (intervention: +0.65; control: +0.26), though this change was not statistically significant (*p* = .586).

Gordon et al. (24) reported mean STAI scores averaged over the post-intervention period up to six months of 33.9 for the MBSR group (reduced from baseline of 40.0) and 39.3 for the waitlist control group (increased from baseline of 36.8), with a reported effect size of Cohen's d = 0.53 (*p* < 0.001).

Huang et al. (31) observed reductions in GAD-7 scores for the MBSR group from 14.39 to 8.13, compared to reductions in the active control group from 14.28 to 11.26 (*p* < 0.001).

Wong et al. (28) did not report immediate post-intervention outcomes. At six-month follow-up (week 32 from enrollment), MBSR group scores on the GCS Anxiety Component were 5.97 (reduced from baseline of 8.23), while active control group scores were 6.97 (reduced from baseline of 8.00). The between-group difference at follow-up was −0.99 (95% CI: −1.71 to −0.28, Cohen's *d* = 0.46), which was statistically significant in complete case analysis (*p* = 0.007) but not in intention to treat (ITT) analysis with imputed data (*p* = .070).

In summary, three of four studies reported statistically significant reductions in anxiety symptoms favoring MBSR groups compared to control groups (24, 25, 31), all *p* ≤ 0.005.

### 3.7 Result outcomes - depression

As a secondary outcome, two studies examined depressive symptoms using different measurement instruments (Table 7), which limits direct numerical comparison of results across studies. Both studies used MBSR interventions, but with different control conditions.

Gordon et al. (24) measured depressive symptoms using the CES-D at baseline (MBSR: 8.9; waitlist control: 10.1) and reported mean scores averaged over the post-intervention period up to 6-month follow-up, weeks 8–32 from enrollment (MBSR: 9.1; waitlist control: 12.4), with a reported effect size of Cohen's *d* = 0.34 (*p* < 0.001). The study did not report immediate post-intervention outcomes.

Wong et al. (28) did not report immediate post-intervention outcomes. At six-month follow-up, MBSR group scores on the GCS Depression Component were 4.78 (reduced from baseline of 6.96), while active control group scores were 5.39 (reduced from baseline of 6.59). The between-group difference was statistically significant (*p* = .031, Cohen's *d* = 0.24).

Both studies assessed depressive symptoms at long-term follow-up (six months, week 32 from enrollment) rather than immediately post-intervention and found statistically significant between-group differences favoring MBSR.

### 3.8 Risk of bias

Risk of bias assessment revealed substantial methodological concerns. Among eight RCTs assessed using RoB 2, none achieved low risk: seven were high risk, and one had some concerns. Two quasi-experimental studies evaluated using the NOS received fair quality ratings (five stars out of nine).

#### 3.8.1 Study design and conduct

Most RCTs demonstrated adequate random sequence generation and baseline comparability, though four provided insufficient detail regarding allocation concealment. One study (31) presented conflicting information about randomization methods. The two quasi-experimental studies lacked randomization and employed two-center designs with geographically separate recruitment sites, introducing potential systematic location-based confounding. All studies delivered interventions in line with established MBI protocols without reported deviations and employed appropriate analyses (ITT or modified ITT). Attrition ranged from 0% to 32%, with two quasi-experimental studies exceeding 20% attrition, raising concerns about potential attrition bias.

#### 3.8.2 Measurement and reporting

All studies relied exclusively on unblinded self-reported questionnaires. Only two studies employed active control conditions providing comparable attention (28, 31); the remainder used waitlist or no-treatment controls. Four RCTs and one quasi-experimental study reported prospective trial registration, though most lacked detailed prespecified analysis plans. Only two studies demonstrated clear protocol adherence (24, 25).

Follow-up duration varied from two weeks post-intervention to six months, with most studies assessing follow-up outcomes at two to four months.

## 4 Discussion

### 4.1 Findings and interpretation

#### 4.1.1 Principal findings and clinical context

This systematic review examined mindfulness-based interventions for mental health during natural menopause across 10 controlled trials involving 1,270 women from six countries. An exploratory random-effects meta-analysis of the primary outcome - psychosocial quality of life, assessed using the MENQOL Psychosocial Domain across six studies - yielded a large pooled effect favouring MBIs [Hedges' *g* = −1.06, 95% CI (−1.45, −0.68), *p* < .001]. Sensitivity analyses demonstrated that this effect was robust across study designs, scoring approaches, and leave-one-out analyses. Anxiety and depression, examined as secondary outcomes using independently administered instruments, showed smaller effects (Cohen's *d* = 0.46–0.53 and 0.24–0.34, respectively). Although these outcomes could not be synthesised quantitatively owing to measurement heterogeneity (four different anxiety instruments, two different depression instruments), the consistency of effect direction across diverse methodological approaches provides some reassurance that observed benefits are not solely dependent on specific instruments or protocols. The substantially larger composite psychosocial effect compared with standalone mood effects raises important questions about what drives the observed benefits, discussed further in Section 4.1.2. While immediate post-intervention benefits were generally consistent, follow-up data revealed variable maintenance of effects, suggesting the potential need for ongoing practice or refresher sessions.

While hormone therapy remains effective for many women, concerns about breast cancer, stroke, and thromboembolic risks (10, 11) have prompted increased interest in alternative treatments. MBIs offer several advantages: they are low-risk, adaptable to various delivery formats, and empower women with self-management skills. The large pooled effect on psychosocial quality of life (*g* = −1.06) suggests MBIs may provide meaningful symptom relief for women experiencing the menopausal transition, given the substantial impact of menopausal symptoms on quality of life, work productivity, and healthcare utilization (7, 9).

#### 4.1.2 The MENQOL psychosocial domain: composition and implications

The observation that composite MENQOL Psychosocial effects exceeded standalone anxiety and depression effects may reflect several factors. As described in Section 3.4, the Psychosocial Domain predominantly captures mood-related symptoms, with two of seven items addressing cognitive difficulties. The discrepancy may therefore reflect differences in measurement properties between menopause-specific and generic psychiatric instruments, or it may indicate that cognitive items contribute to the observed improvement. These interpretations are not mutually exclusive, and current evidence cannot distinguish between them.

Even if cognitive items do contribute to the observed psychosocial improvement, it remains unclear whether this reflects genuine changes in cognitive capacity or changes in how women perceive their cognitive difficulties when emotional distress is reduced. No included study employed validated cognitive assessment measures, such as neuropsychological testing or standardised cognitive complaint scales, despite evidence that subjective cognitive difficulties are commonly reported during menopause (5, 6) - making it impossible to distinguish between these possibilities.

Nevertheless, the consistency of psychosocial improvements across varied intervention formats, menopausal stages, and cultural settings suggests that the cognitive components within psychosocial outcomes warrant dedicated investigation. This question has particular clinical relevance because, unlike menopausal anxiety and depression, there are currently no established treatment options specifically targeting subjective cognitive complaints during menopause; although hormone replacement therapy has been explored for cognitive symptoms, evidence remains inconsistent and it is not currently recommended for this indication (35). Given that mindfulness can enhance attentional control and working memory (36), future studies incorporating validated cognitive outcome measures are needed to clarify whether cognitive symptom experiences represent a meaningful component of psychosocial improvement during menopause.

#### 4.1.3 Durability of intervention effects

Evaluating durability is complicated by methodological inconsistencies and substantial variation in follow-up length (two weeks to six months). Durability can be conceptualized as maintenance of initial gains (post-intervention to follow-up) or sustained benefit over baseline (follow-up vs. baseline relative to controls). From both perspectives, psychosocial symptoms appear relatively more durable: all studies with follow-up data showed sustained benefits over baseline, and only one of five studies showed partial degradation from post-intervention levels. In contrast, anxiety outcomes showed more variable patterns. From a sustained benefit perspective, most studies maintained improvements over baseline at follow-up. However, from a maintenance of gains perspective, several studies lost statistical significance between post-intervention and follow-up assessments despite participants remaining improved from baseline. The reasons for greater psychosocial durability compared with anxiety outcomes are unclear and may reflect measurement properties, symptom trajectories, or differential practice effects. However, the degradation of anxiety benefits over time suggests that mindfulness benefits may depend on ongoing practice. This aligns with mindfulness theory emphasizing sustained practice, though no studies tracked post-intervention adherence or tested refresher session protocols.

Depression outcomes require particular caution in interpretation, as both studies assessed symptoms only at six-month follow-up without immediate post-intervention data, precluding assessment of maintenance patterns. Notably, Gordon et al. (24) found that while MBSR did not reduce existing depressive symptoms, it attenuated symptom worsening compared to controls over six months (*p* < .001, *d* = 0.34). This protective pattern suggests MBIs may help maintain mood stability during the menopausal transition - buffering against mood deterioration during a period of increased vulnerability - even when they do not produce immediate symptom relief.

### 4.2 Limitations

#### 4.2.1 Evidence quality and risk of bias

The most significant methodological limitation concerns the overall quality of the evidence base, as no studies achieved low overall risk of bias. All studies relied exclusively on self-reported questionnaires, and since participants were necessarily aware of their group allocation - an inherent limitation of behavioural intervention research - these outcomes may be susceptible to expectancy and reporting bias. This concern is particularly relevant given that only two of ten studies employed active control conditions providing comparable attention and time commitment; the remainder used inactive controls where expectancy effects are most pronounced. Nevertheless, the pooled MENQOL Psychosocial effect remained large and statistically significant across all sensitivity analyses, suggesting that the observed benefits are unlikely to be entirely attributable to methodological artefact, though the true effect magnitude may be smaller than the pooled estimate indicates.

#### 4.2.2 Measurement heterogeneity

Measurement heterogeneity was pronounced for both anxiety (four different instruments across four studies) and depression (two instruments across two studies), precluding quantitative synthesis of these secondary outcomes. Additionally, inconsistent statistical reporting - with only two studies providing standardised effect sizes - and variation in assessment timing (some studies lacking immediate post-intervention data, follow-up ranging from two weeks to six months) further limited cross-study comparison. As discussed in Section 4.1.2, the absence of validated cognitive assessment measures across all included studies represents a significant limitation, preventing evaluation of whether MBIs influence cognitive function independently of mood-related changes.

#### 4.2.3 Sample representativeness and cultural context

Sample representativeness is a further concern. The demands of eight-week MBI protocols - including weekly group attendance and daily home practice - likely favour women with time flexibility, educational backgrounds conducive to introspection, and resources to prioritise self-care. While studies spanned six countries, several acknowledged recruiting relatively homogeneous samples. Cultural context may also influence outcomes: The study by John and colleagues (20), conducted in India where meditative practices have deep historical roots, reported zero attrition and the largest effect size, raising the possibility that cultural familiarity with mindfulness may enhance engagement and response. Whether MBI benefits generalise across diverse cultural settings and to women facing greater structural barriers remains unclear. Additionally, inconsistent menopause stage classification across studies challenges cross-study comparison. Most included studies did not recruit participants based on formal psychiatric diagnoses or clinically significant symptom thresholds, limiting interpretation regarding the effectiveness of MBIs for diagnosed anxiety or depressive disorders during menopause.

#### 4.2.4 Publication bias

Finally, potential publication bias due to the exclusion of non-English language studies and unpublished data cannot be ruled out. However, of the non-English studies identified in the original search, only one (a Persian version of an included study) met eligibility criteria after title and abstract screening, minimising this risk.

### 4.3 Clinical implications

#### 4.3.1 Clinical utility

The pattern of findings has several clinical implications, though these must be interpreted cautiously given methodological limitations. The simultaneous improvements across psychosocial and mood domains suggest MBIs may be particularly suitable for women experiencing multiple co-occurring mental health symptoms during menopause, potentially reducing reliance on multiple symptom-specific treatments. As self-directed interventions that cultivate autonomy and emotional regulation, MBIs offer a low-risk option for women seeking non-pharmacological approaches or those with contraindications to hormone therapy (21, 24, 28, 30). However, women should be advised that MBIs represent active, skill-building interventions requiring ongoing engagement, as benefit maintenance varied across outcome domains, with anxiety effects showing particular vulnerability to degradation over time.

#### 4.3.2 Implementation considerations

Translating these findings into clinical practice faces practical barriers. Standard eight-week protocols requiring weekly group attendance and daily home practice may not be feasible for women with caregiving responsibilities, work constraints, or limited resources (24, 28). Adapted delivery formats - including shortened protocols or digital approaches - may improve accessibility but require effectiveness validation. Gordon and colleagues' study (24), focusing on women during the transition period, found that MBSR did not reduce existing depressive symptoms but prevented the worsening observed in controls, suggesting that MBIs initiated early in perimenopause may have protective value before symptoms become established, though this hypothesis requires direct testing. Current evidence does not support screening approaches to identify which women will benefit most, and clinical implementation should balance potential benefits against patient capacity and available resources.

### 4.4 Research priorities

#### 4.4.1 Cognitive function assessment

A critical gap in cognitive assessment demands urgent attention. This review identified no studies employing dedicated neuropsychological measures or validated cognitive complaint instruments, despite 44%–62% of menopausal women reporting subjective cognitive difficulties (5, 6). Given the limited evidence for treatments specifically targeting subjective cognitive complaints during menopause, determining whether MBIs improve cognitive symptoms represents an important unmet therapeutic need. Future studies must incorporate comprehensive cognitive assessments to determine whether MBIs produce genuine cognitive benefits and, if so, which cognitive domains (e.g., memory, attention, executive function) are most responsive.

#### 4.4.2 Long-term effectiveness and maintenance

As detailed in Section 4.1.3, benefit maintenance varied across outcomes, with psychosocial improvements showing superior durability while anxiety showed more variable patterns. A critical methodological gap is that no studies tracked post-intervention practice adherence despite theoretical emphasis on sustained practice for maintaining benefits. Future research should investigate the minimum practice dose required to maintain benefits, comparing outcomes between participants maintaining regular practice vs. those who discontinue, and testing whether structured booster sessions can prevent the degradation of effects observed for anxiety outcomes.

#### 4.4.3 Comparative effectiveness

Comparative effectiveness trials directly testing MBIs against established symptom-specific treatments are essential for informing clinical decision-making. No studies compared MBIs with evidence-based interventions such as CBT for anxiety or pharmacological approaches for mental health outcomes. Studies of adapted delivery formats - including shortened protocols or digital approaches - are also needed to improve accessibility for women facing structural barriers and to ensure that evidence reflects real-world effectiveness across diverse populations.

## 5 Conclusion

While research on the effects of MBIs on menopause-related mental health disturbances, including depression and anxiety, remains limited, existing evidence suggests MBIs may improve mental health outcomes in menopausal women, offering a promising non-pharmacological approach to symptom management. Although cognitive effects remain underexplored, due to a lack of dedicated cognitive assessment, the discrepancy between composite psychosocial effects and standalone mood effects may reflect measurement differences between menopause-specific and generic instruments, or suggest that cognitive complaints contribute to the observed benefits. However, these findings should be interpreted cautiously given the substantial methodological limitations across included studies, including high risk of bias, reliance on self-reported outcomes, and the frequent use of inactive control conditions. Future research should prioritise validated cognitive assessment as an independent outcome, investigation of practice dose and treatment duration required to maintain benefits, and comparative effectiveness trials against established treatments. Strengthening this evidence base will be critical for integrating MBIs into clinical practice and expanding safe, sustainable, and accessible treatment options for menopausal women.
