# Supplementary figures and images for: Effectiveness of mindfulness-based interventions on mental health in natural menopause: a systematic review and meta-analysis
Source: Front Glob Womens Health. 2026 Jul 10;7:1830813. doi: 10.3389/fgwh.2026.1830813 (PMC13396212; doi:10.3389/fgwh.2026.1830813)

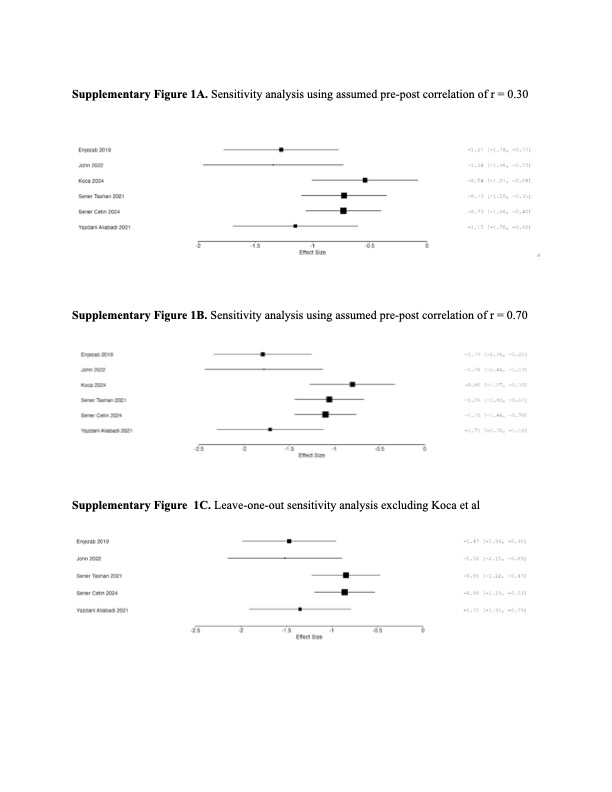

Supplement: Supplementary file 1 [file Image1.jpeg]

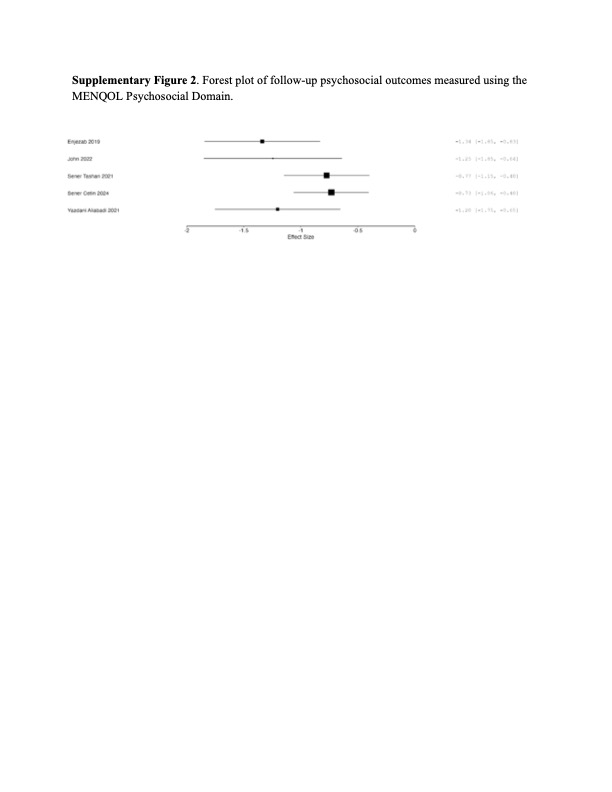

Supplement: Supplementary file 2 [file Image2.jpeg]
